# Supplementary material for: Hepatotropic Peptides Grafted onto Maleimide-Decorated Nanoparticles: Preparation, Characterization and In Vitro Uptake by Human HepaRG Hepatoma Cells
Source: Polymers (Basel). 2022 Jun 16;14(12):2447. doi: 10.3390/polym14122447 (PMC9229302; doi:10.3390/polym14122447)

polymers

MDPI

Article

Supplementary Materials: Hepatotropic peptides grafted onto maleimide-decorated nanoparticles: preparation, characterization and *in vitro* uptake by human HepaRG hepatoma cells

Clarisse Brossard[1], Manuel Vlach[2,3], Lucas Jacquet[1], Elise Vène[2,4], Vincent Dorcet[1], Pascal Loyer[2,*], Sandrine Cammas-Marion[1,2,*], Nicolas Lepareur[2,5,*]

Figure S1: DLS size distribution report by intensity of: A. NPs 1′, B. and B. NPs 7′.

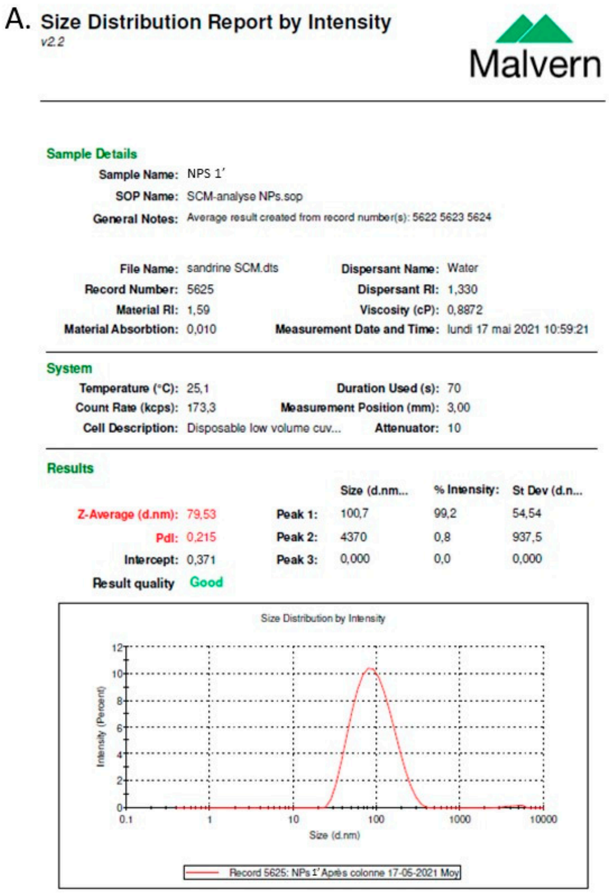

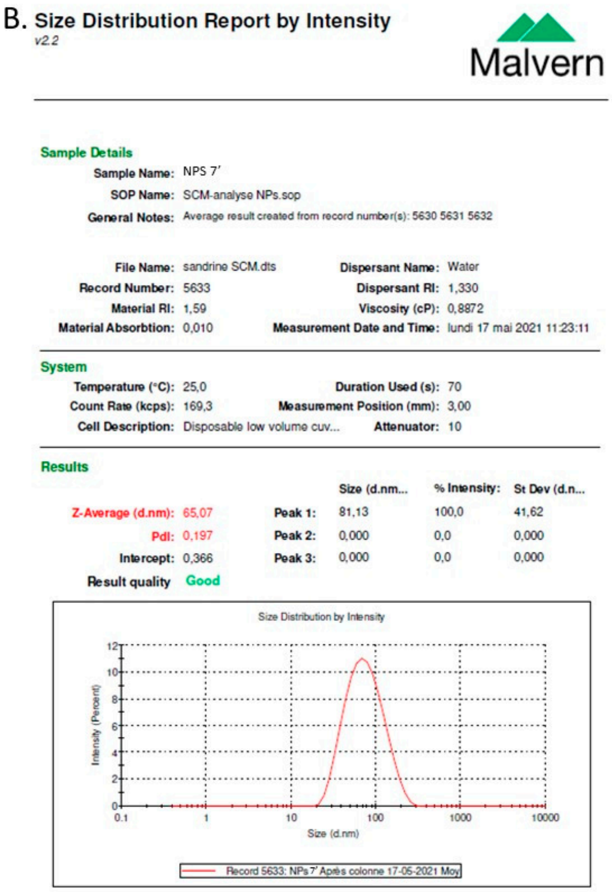

Figure S2: 1H NMR spectra of: A. PMLABe$_{73}$, B. PEG$_{42}$-*b*-PMLABe$_{73}$, C. Mal-PMLABe$_{73}$, and D. Mal-PEG$_{62}$-*b*-PMALBe$_{73}$.

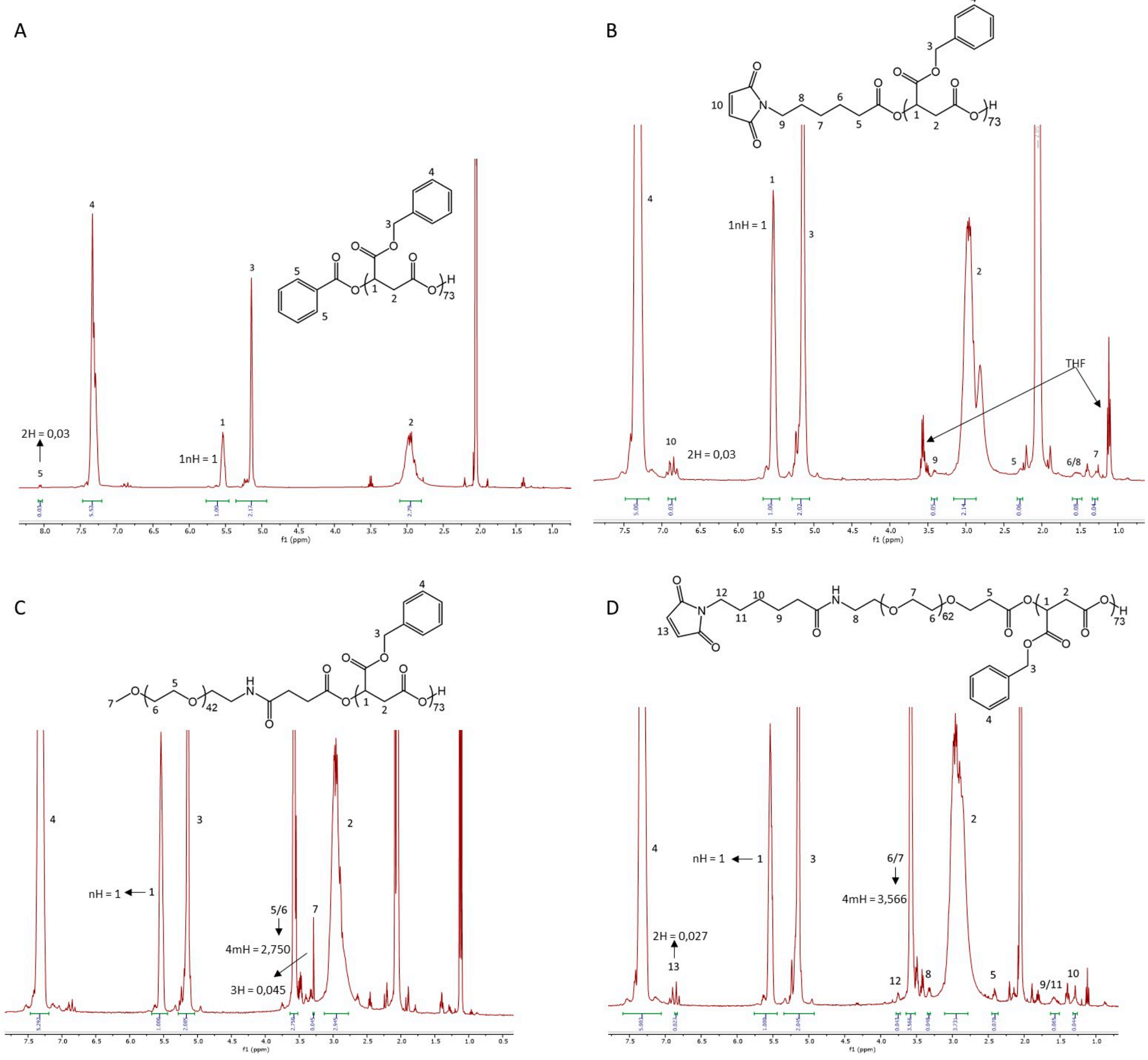

Figure S3: SEC (THF, 40°C, Polystyrene standards) of: A. PMLABe$_{73}$, B. PEG$_{42}$-*b*-PMLABe$_{73}$, C. Mal-PMLABe$_{73}$, and D. Mal-PEG$_{62}$-*b*-PMALBe$_{73}$.

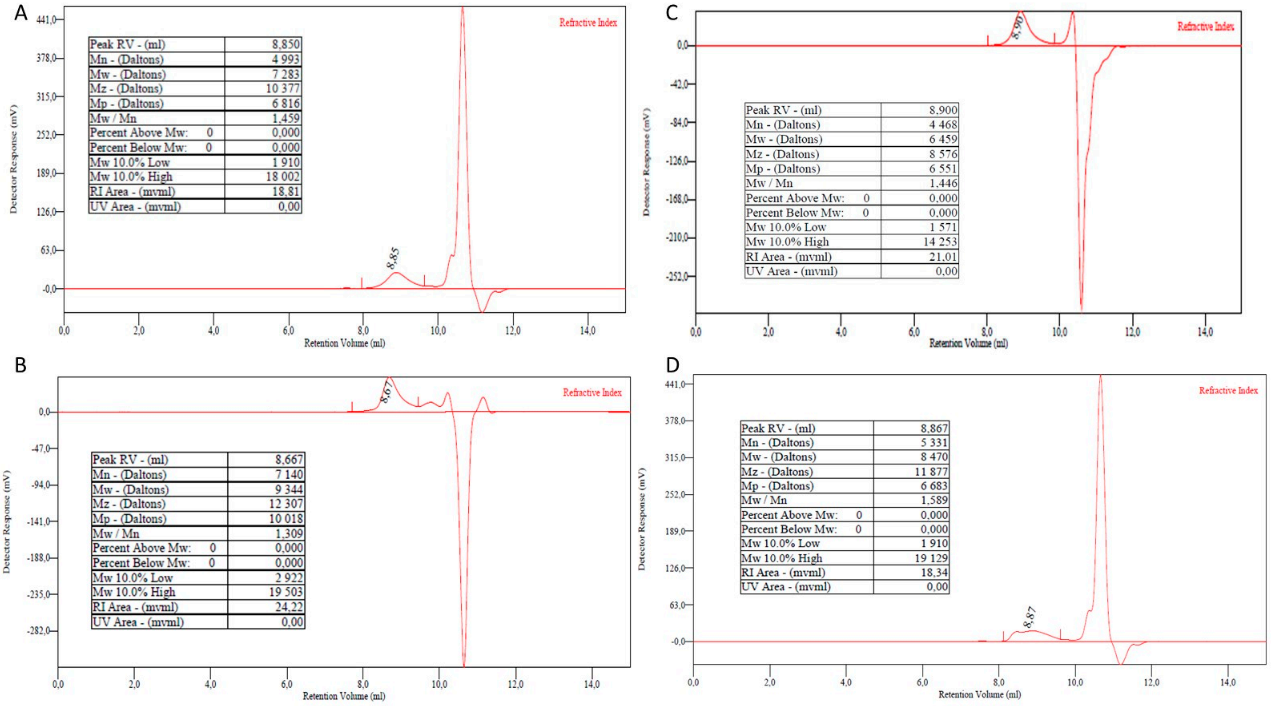

Figure S4: DSC thermograms of: A. $PMLABe_{73}$, B. PEG_{42}-*b*-$PMLABe_{73}$, C. Mal-$PMLABe_{73}$, and D. Mal-PEG_{62}-*b*-$PMALBe_{73}$.

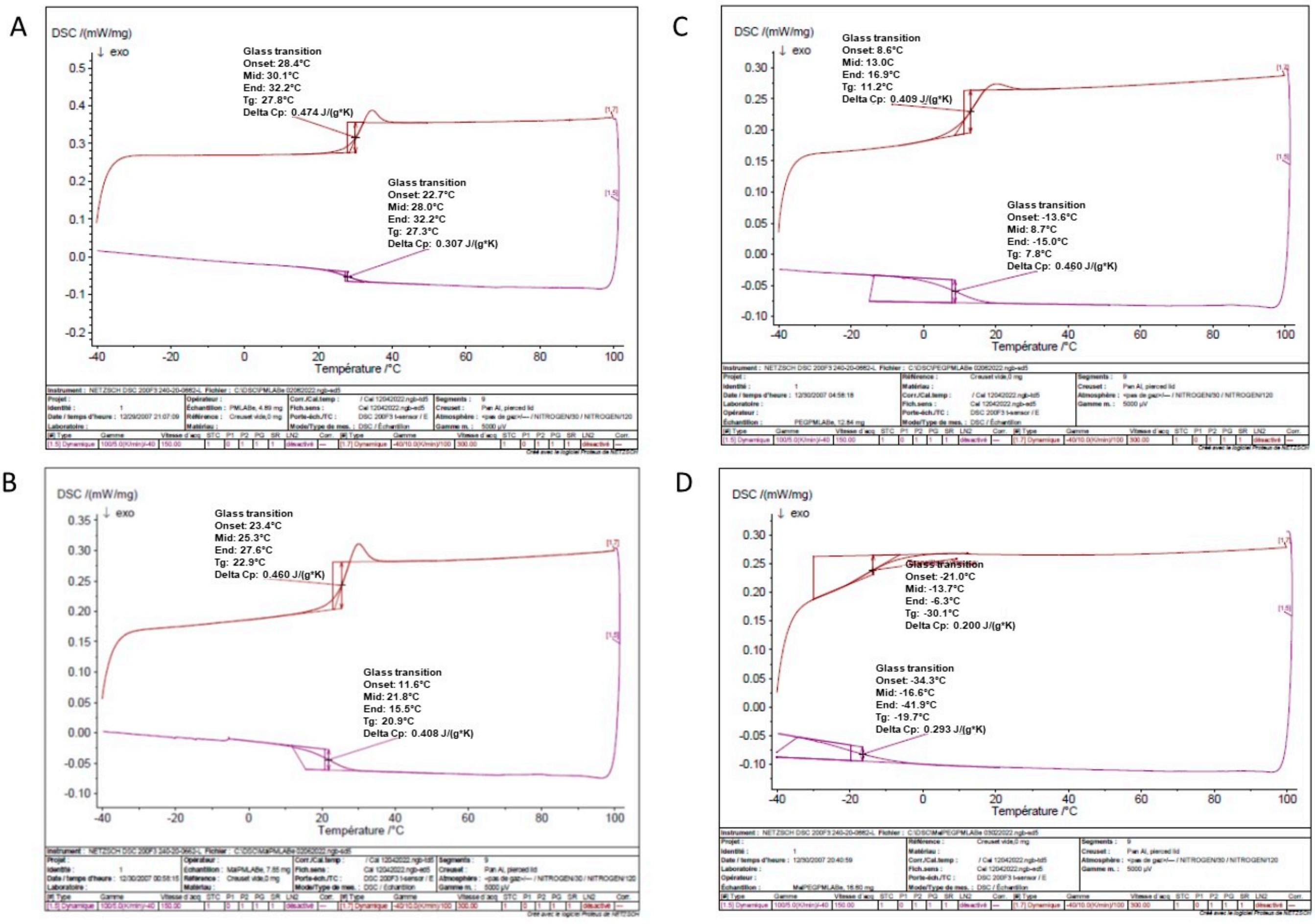

Figure S5. A. Diameters (Dh) and B. dispersity (PDI) of NPs obtained by the post- and pre-formulation methods.

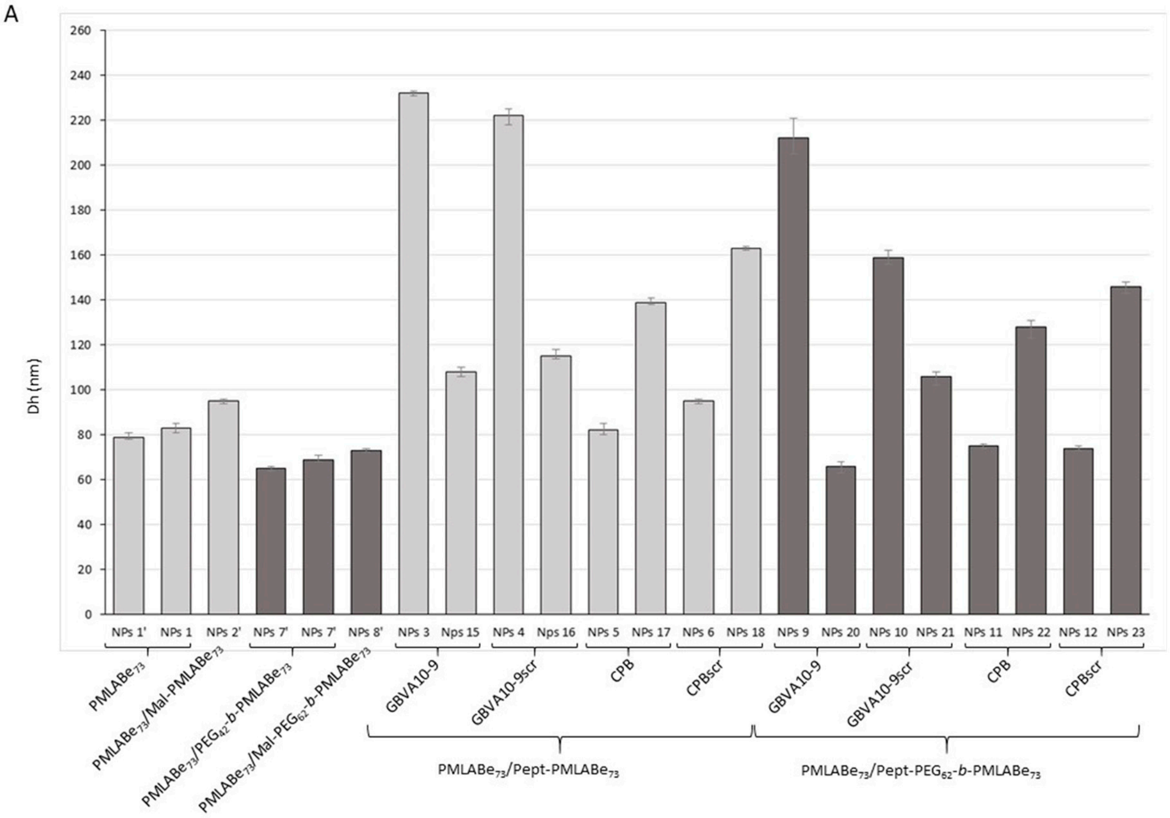

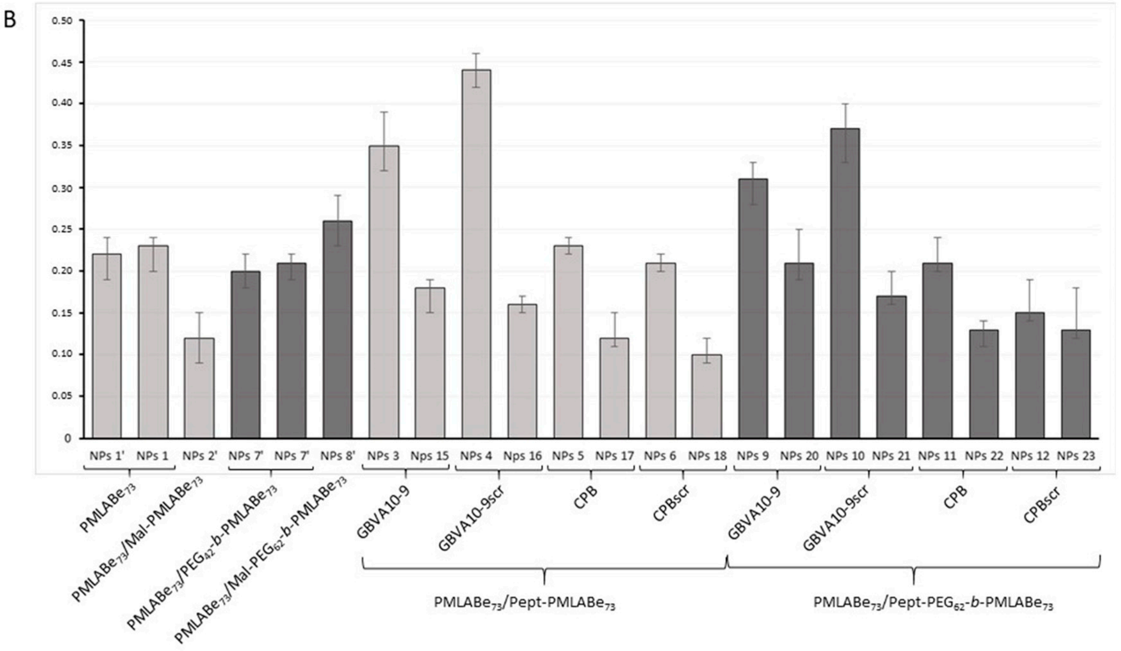

Supplement: Supplementary file 1 [file polymers-14-02447-s001.zip › polymers-1742771-supplementary.pdf]
